# Improved sustainability of solar panels by improving stability of amorphous silicon solar cells

**DOI:** 10.1038/s41598-023-37386-5

**Published:** 2023-06-29

**Authors:** Gautam Ganguly

**Affiliations:** GG Consulting, 4435 N Dryden Street, Prescott Valley, AZ 86314 USA

**Keywords:** Energy science and technology, Materials science

## Abstract

As the world grapples with global warming, it becomes imperative to carefully examine the sustainable energy technology choices. Solar is the fastest growing clean energy source but today it contributes little to the electricity generated, so future installations will dwarf the existing installed base. There is a factor of 2–4 decrease in the energy payback time from the dominant crystalline silicon technology to thin film technologies. Essential criteria like use of abundant materials and simple but mature production technology point to amorphous silicon (a-Si) technology. Here we delve into the primary issue impeding adoption of a-Si technology—the Staebler Wronski Effect (SWE), that generates metastable, light induced defects which reduce the performance of a-Si based solar cells. We demonstrate that a simple change leads to a significant reduction in SWE power loss and define a clear path to elimination of SWE, allowing the technology to be widely adopted.

## Introduction

Solar cells are an important component of the sustainable energy mix required to contain global warming and the move to electricity based rather than a fossil fuel based economy. While cumulative solar installations aggregate 1.2 TW^1,2^ this contributes less than 5% of the electricity generated worldwide^3^. Clearly, the immensity of future installations will dwarf the current installed base. It therefore behooves us to take a careful look at our technology choices for the future. Since the current energy use is dominated by fossil fuels, the energy required to produce solar panels is very important in the contribution it makes to global warming. This energy requirement needs to be compared to the energy generated when the solar panel is deployed, and this is captured in the EPBT^4,5^. EPBT is reduced by the specific yield (SY = energy generated in the field/ power output under standard condition/) of the solar panels which captures the standard power rating system used for solar panels that is measured at 25 C and 1000 W/m^2^ irradiance, and the energy generated by the solar panel in the field, which is impacted by the actual operating temperature that is typically much higher than 25 C (about 40 C on average) as well as the irradiance which is typically lower than 1000 W/m^2^ (200–800 W/m^2^). Comparison of different commercially available technologies reveal that thin film technologies have lower EPBT^4,5^. This accrues from the smaller temperature dependence of power^6^ and hence generate more energy at the higher than 25 C operating temperatures and they also have a smaller irradiance dependence of power output^7^ resulting in more energy generation at the lower than 1000 W/m^2^ irradiance in the field. While the industry has largely ignored these realities because the power output under standard (STC) conditions is easier to measure, technology based EPBT^4,5^ and SY^7^ can be determined and is significantly lower (2–4 times) for thin film technologies compared to the crystalline silicon technology that constitutes 95% of the industry currently.

Of the thin film technologies, a-Si has the advantage of the highest SY and uses abundant materials, requires the lowest manufacturing temperatures (about 200 C) and the thinnest semiconductor layers (less than 5000 nm)^7,8^. It suffers from one fatal issue—the so-called SWE^9^ which limits its efficiency to 13% for three junction solar cells^10,11^. However, 16% three junction solar cells have been demonstrated, before SWE^10,11^, and over 20% four junction solar cells have been projected, in the absence of SWE^12^. a-Si technology has already been proven in commercial volume production by numerous companies. The improvement of efficiency in the absence of SWE would put this technology in the very short EPBT range relative to other technologies^7^ and make it the first choice for sustainability.

Current best performance a-Si solar cells use a so-called hydrogen dilution method^13^ that reduces SWE, but single junction cells deposited at about 0.1 nm/s still exhibit about 20% SWE power losses while two and three junction cells have smaller losses^13^. It is well known that substrate temperature is a significant factor in the amount of SWE performance loss in a-Si based solar cells^14^. However, we have shown that it is not the substrate temperature but the chamber or gas temperature that determines the SWE performance loss^14^. This can be understood in terms of the polysilane based origins proposed for the SWE defects^15^ and suggests that reactions that generate polysilanes in the silane (SiH_4_)-hydrogen (H_2_) plasmas used to deposit a-Si, are exothermic as has been verified in several independent studies^16^. Here, we use this information to shift control of the gas temperature from heating of the anode to heating of the earth-shield, that surrounds the cathode, which allows us to control SWE independently from the substrate temperature.

## Results and discussion

**Figure 1:**
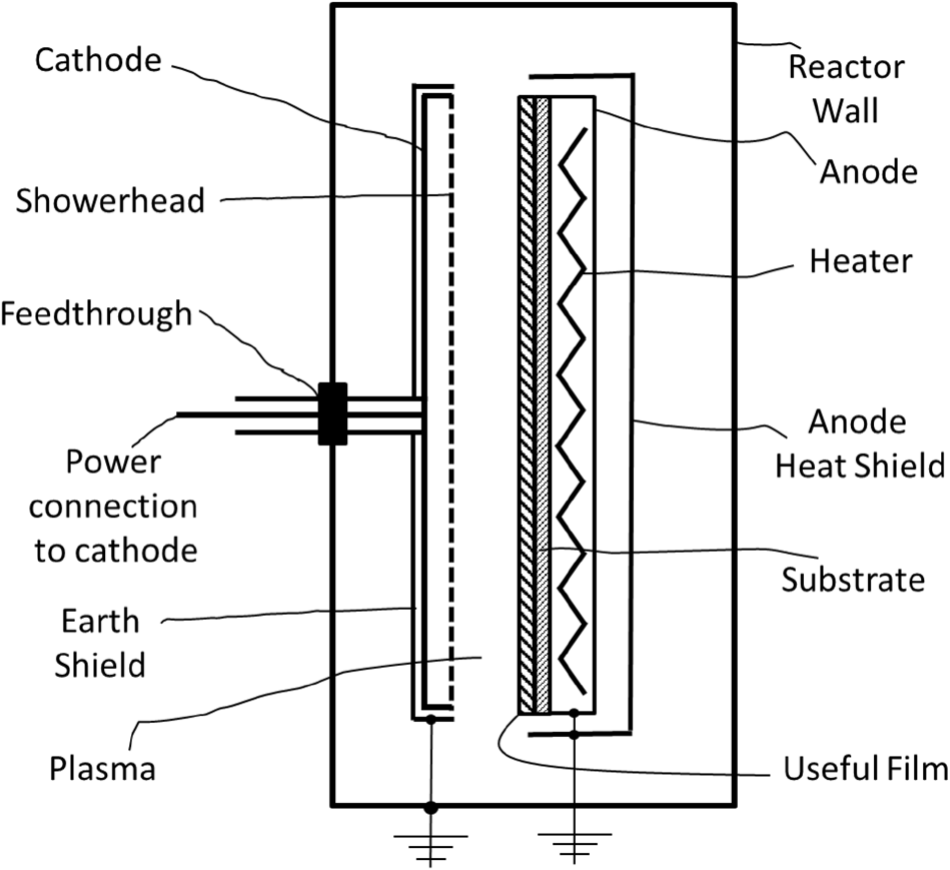
Schematic outline showing different parts of a standard plasma reactor used for deposition of aSi based films and solar cells. Not to scale.

In Fig. 1 we see a schematic of a standard parallel plate, plasma enhanced, chemical vapor deposition reactor that is used in fabrication of a-Si based films, solar cells and modules. The substrate is heated from a heater placed in the anode while the reactive gases and plasma generation power (DC or RF) is supplied to the cathode. An earth-shield placed close to (less than the ionization length) around the cathode prevents formation of plasma between cathode and reactor walls, and thus confines the plasma to the space between the cathode and anode. This results in useful film deposition on the substrate placed in thermal contact with the heated anode. In this configuration, the cathode is radiatively and conductively heated from the anode. When optimum substrate temperatures of ~200 °C are used the cathode temperature is between 75 and 180 °C depending on the spacing between the cathode and anode as well as the gas pressure and power supplied to the cathode.

**Figure 2:**
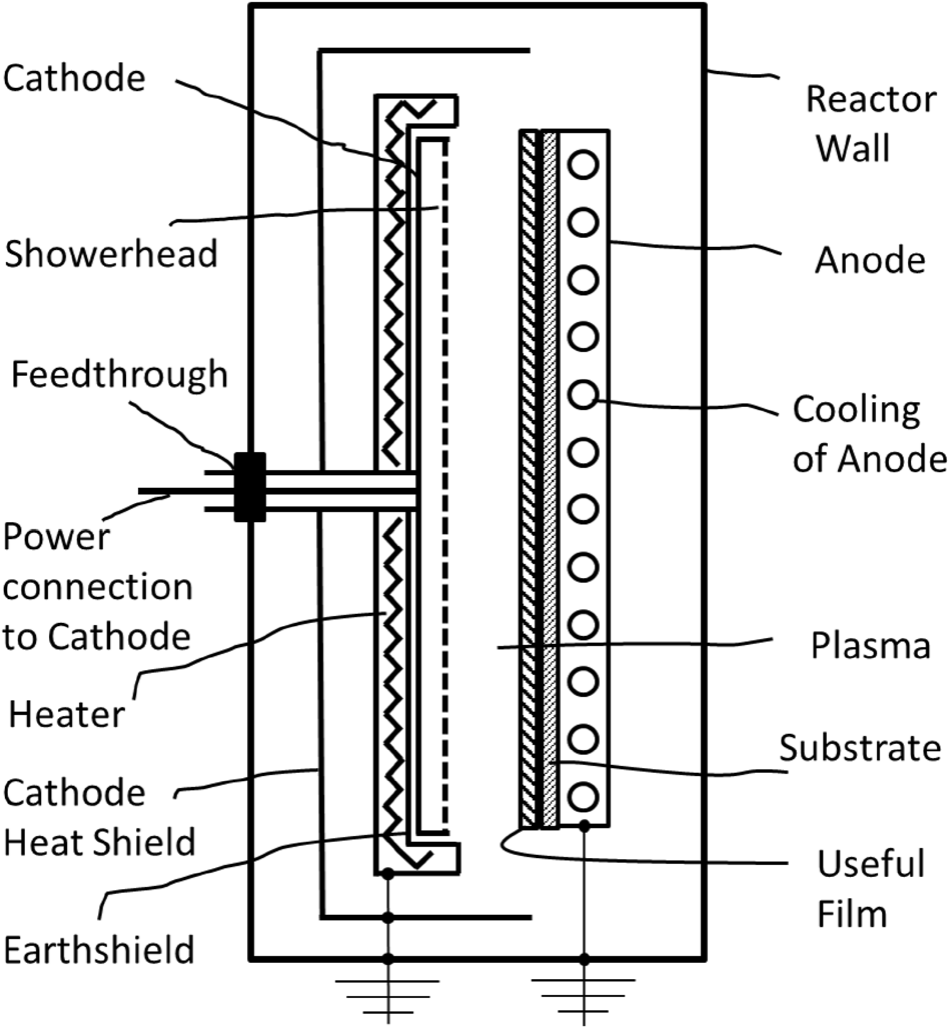
Outline showing different parts of proposed plasma reactor to used for deposition of aSi based films and solar cells exhibiting little to no SWE. Not to scale.

In Fig. 2 a different reactor configuration is proposed which incorporates the heater in the earth-shield surrounding the cathode while the anode is cooled (using liquid or gas) to obtain a suitable temperature on the substrate. In the present study, the substrate temperature is held at the same value of 200 °C but no cooling is used for the anode. The temperature at the earth-shield is about 350° C. Cooling the anode would allow higher temperatures to be obtained at the earth-shield and improved results are expected to be obtained.

**Figure 3:**
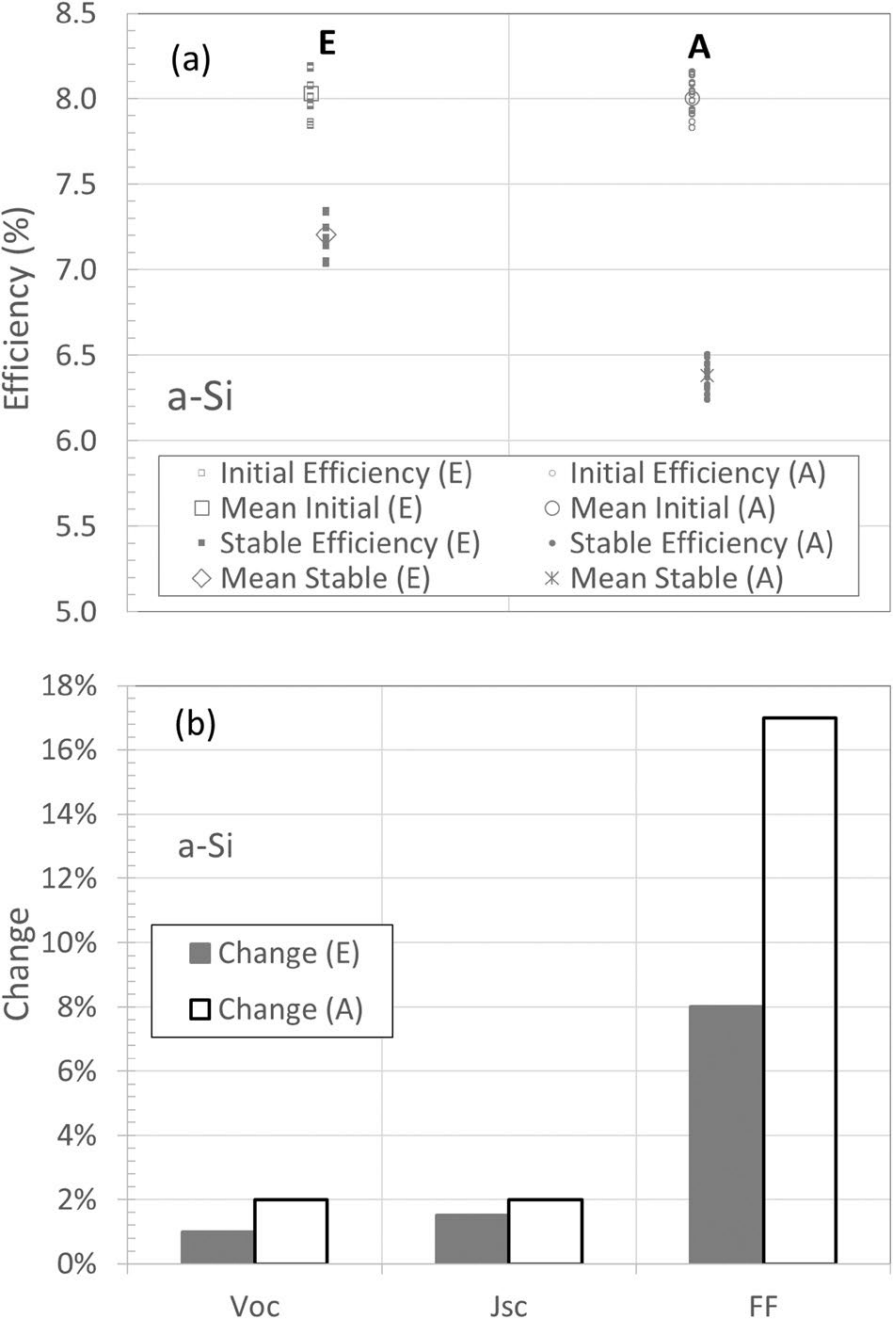
(**a**) The initial and stable efficiency of amorphous silicon solar cells deposited at a substrate temperature of 200 C using heating of the earth-shield (E) or conventional heating of the anode (A). Mean values of the efficiency of each group of cells are also indicated. (**b**) The change in the average values of the open circuit voltage (V_oc_), short circuit current density (J_sc_) and the fill Factor (FF) for the same cells as in (**a**).

The results presented here^17^ are for single junction a-Si and dual (tandem) junction silicon/silicon–germanium (a-Si/a-SiGe) solar cells deposited on low cost, commercially available, tin oxide coated 3 mm thick soda lime glass using a parallel plate, DC plasma enhanced chemical vapor deposition system. The gas used was silane-hydrogen mixture for a-Si intrinsic layers and silane-germane(GeH_4_)-hydrogen gas mixture for a-SiGe intrinsic layers. The deposition rate for both intrinsic layers was about 0.1 nm/s. In Fig. 3a we show the initial and stable (after 600 h light illumination with 100 mw/cm^2^ at 50 °C) efficiency of two sets of a-Si cells deposited with either, as is conventionally used, the anode (A) *or* in this case, the earth-shield (E) is heated, to maintain a temperature of 200 °C at the substrate. Figure 3b shows the relative change in the average solar cell performance parameters after light soaking for the same cells as in Fig. 3a.

**Figure 4:**
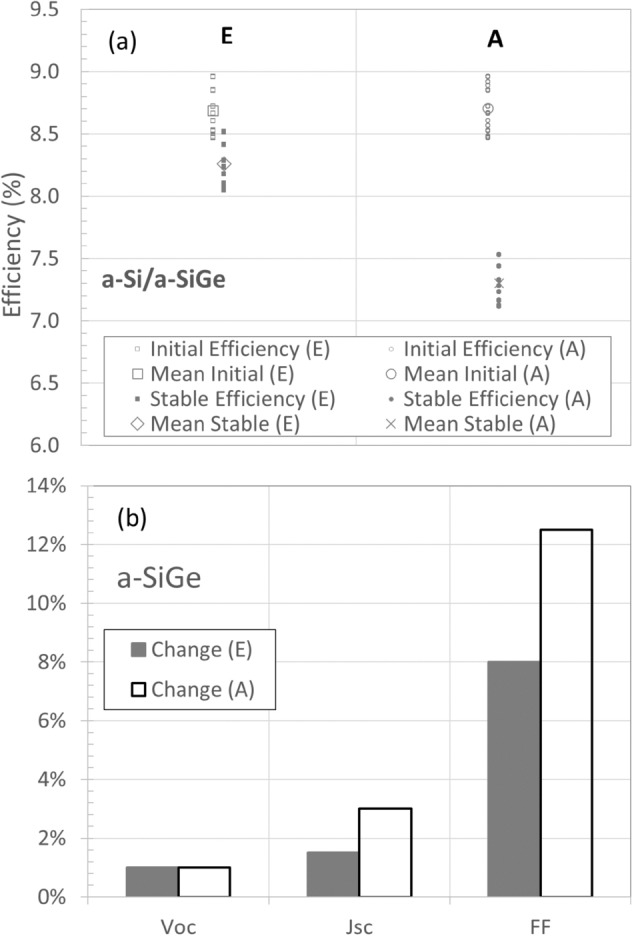
(**a**) The initial and stable efficiency amorphous silicon/silicon germanium solar cells deposited at a substrate temperature of 200 °C using heating of the earth-shield (E) or conventional heating of the anode (A). Mean values of the efficiency of each group of cells is also indicated. (**b**) The change in the average values of the open circuit voltage (V_oc_), short circuit current density (J_sc_) and the fill Factor (FF) for the same cells as in (**a**).

While Fig. 3a,b show the results for a-Si single junction cells, Fig. 4a,b show results for a-Si/a-SiGe tandem junction cells. The initial efficiency is similar but the stable efficiency is higher for the earth-shield heated (E) samples than the anode heated (A) samples. Each data point is an average of fifteen 0.25 cm^2^ cells on each of two 10 cm^2^ pieces cut from a 30 cm × 30 cm piece of glass substrate used for each deposition run. At least 10 deposition runs were used for each heating mode (A or E) and each type of cell (a-Si and a-Si/a-SiGe). All cells were annealed at 160 °C for 1 h after light soaking and retested. At least one additional round of light soaking followed by annealing was carried out to assure reversibility of SWE, and reproducibility of the results.

In a parallel plate, plasma enhanced chemical vapor deposition reactor, typically used for deposition of a-Si based solar cells and modules, the source gas silane is primarily dissociated through energetic electron dissociation^18^ following the pathways (1) and (2) forming silylene (SiH_2_) and silyl (SiH_3_) radicals, respectively,

1 $${\text{SiH}}_{4} + {\text{ e}}* \, = {\text{ SiH}}_{2} + {\text{ H}}_{2} - \Delta {\text{H}}$$

2 $${\text{SiH}}_{4} + {\text{ e}}* \, = {\text{ SiH}}_{3} + {\text{ H}} - \Delta {\text{H}}$$

The formation of the primary polysilane (Si_n_H_2n + 2_), disilane (Si_2_H_6_), occurs through the secondary reaction between silane and silylene,

3 $${\text{SiH}}_{2} + {\text{ SiH}}_{4} = {\text{ Si}}_{2} {\text{H}}_{6} + \Delta {\text{H}}$$

Similarly, trisilane (Si_3_H_8_) is formed by the reaction between disilane and a silylene radical,

4 $${\text{SiH}}_{2} + {\text{ Si}}_{2} {\text{H}}_{6} = {\text{ Si}}_{3} {\text{H}}_{8} + \,\Delta {\text{H}}$$

And in general,

5 $${\text{SiH}}_{2} + {\text{ Si}}_{n} {\text{H}}_{2n + 2} = {\text{ Si}}_{n + 1} {\text{H}}_{2n + 2} + \Delta {\text{H}}$$

These secondary reactions are exothermic^16^ and thus need a third body to remove the excess thermal energy. Since silane and the electrical power used to excite electrons are fed into the reactor through a showerhead in the cathode, the highest density of silylene occurs close to the cathode. Given the exothermic nature of these reactions one sees that to reduce its formation, one needs to focus on the region close to the cathode. The earth-shield temperature is typically about 150 °C in the ‘A’ configuration of Fig. 1 while heating the earth-shield to 350 °C in the ‘E’ configuration of Fig. 2 would be expected to reduce the rate of reaction 3 by 7 orders of magnitude based on the activation energy in reference 16. This is reflected in the results for a-Si cells in Fig. 3. We have demonstrated in a standard configuration reactor, as illustrated in Fig. 1, that the stability of the a-Si solar cells deposited at the center is improved when the temperature uniformity from center to the edge of substrate is improved^19^. This was suggested to be due to polysilane species forming faster when the edge is cooler and diffusing towards the center. This is also consistent with the exothermic nature of reaction 3.

The results of Fig. 4 further show that the suppression of SWE occurs in a-SiGe cells as well. a-SiGe cells exhibit greater SWE than a-Si cells and in a tandem cell, the a-SiGe cell determines the degree of SWE^8,13^. One can infer from these results that increasing the earth-shield temperature to even higher values (350–600 °C) using a reactor of the configuration illustrated in Fig. 2, would further suppress the formation of polysilane species and potentially eliminate SWE in a-Si. This would allow re-optimization of a-Si based solar cells to yield efficiencies of 20% or more^12^.

The current results demonstrate that SWE, caused by polysilane species formation near the earth shield and cathode, can be suppressed by heating the earth-shield instead of the anode to achieve the desired substrate temperature, as demonstrated by the results in Fig. 3 and Fig. 4. Cathode heating has been previously investigated^20,21^ where the anode and cathode were independently heated to as high as 300 °C. It was observed that the hydrogen content, that is determined by the bulk temperature of the film, was controlled by the anode temperature while the defect density, which is determined by the temperature of the growth precursors at the growth surface^22^, was impacted by the cathode temperature. The advantage of having a *higher* temperature at the cathode *and the earth shield* while maintaining the optimum substrate temperature by *not heating* the anode or even cooling the anode as illustrated by the apparatus of Fig. 2, for reduction of SWE, was not realized.

Understanding and controlling the source of polysilane species formation has other implications as well. It is not known if the earth-shield heating method would reduce SWE in carbon or oxygen-based alloys of a-Si that are typically used to make wide band gap solar cells with higher Voc. However, it is well known that lower substrate/deposition temperatures that lead to wide band gap a-Si cells potentially having higher V_oc_ unfortunately come with increased defect density as well, which results in lower FF and poor initial efficiency. When the deposition temperature is reduced below the optimum value of 200 °C, the earth-shield and cathode also cool and the density of polysilane species derived radicals in the plasma increase, and their contribution to deposition is likely responsible for the poorer initial quality of the film. This can be easily verified by heating the earth-shield independently to 350–600 °C while cooling the anode to maintain a substrate temperature *lower than* 200 °C. This would result in high quality (high FF) a-Si solar cells with a wide band gap (high V_oc_). Such solar cells would also be expected to have low SWE and potentially be incorporated in 4 junction a-Si solar cells.

In preparation of a-Si cells at higher deposition rates, the power density applied to the cathode is increased and this generates increased density of silyl and silylene radicals near the cathode and earth shield. Therefore, the current method of earth-shield heating would also allow maintaining low SWE when the deposition rate of a-Si is increased in a-Si based solar cells. In case of a-SiGe, it is known that polygermane forms more rapidly relative to polysilane as observed from higher rates of particulate formation^23^. It was shown that increasing the substrate temperature reduced the particulate density^23^. This implies that the lower film quality of a-SiGe relative to a-Si is, to some extent, associated with the higher rate of polygermane formation and its larger contribution to film formation. The effect of cathode heating was shown to reduce the defect density in a-SiGe films when the cathode temperature was increased from unheated to 300 C with the substrate temperature set at 150 C^20^. This suggests that using the earth-shield heating technique (instead of anode heating) will yield higher quality a-SiGe films, which would contribute to even higher efficiencies of a-Si based multijunction films. Higher anode temperatures are used to make higher quality a-SiGe solar cells, which works well for n–i–p type cells^13^, where the a-SiGe cells are deposited first, but not for the more common p–i–n cells where the a-SiGe cells are deposited later^8,13^. Using the earth-shield heating method would improve the quality and hence, performance of a-SiGe cells even at lower substrate temperatures.

The benefits of earth-shield heating may hold true for amorphous silicon carbon alloys and silicon oxygen alloys used for making wide band gap alloys which suffer from severe SWE and have not typically been used as intrinsic layers in high efficiency a-Si based multijunction solar cells. Finally, SWE free amorphous alloys of silicon would void the need for microcrystalline or nanocrystalline silicon as the smaller optical bandgap cells in multijunction solar cells. This would be a significant benefit as the microcrystalline material has a lower deposition rate and require greater thickness in the solar cells due to its indirect optical absorption, similar to crystalline silicon.

## Conclusions

It has been demonstrated that heating the earth-shield (rather than heating the anode as is conventionally done), in a parallel plate plasma chemical vapor deposition system for making a-Si and a-Si/a-SiGe solar cells at a substrate temperature of 200 °C, significantly reduces SWE in a-Si and a-SiGe solar cells. A system configuration is proposed that can potentially reduce SWE in a-Si based devices to insignificant levels (< 2%). It is suggested that this happens due to the reduction of polysilane species formation that occur dominantly through exothermic reactions close to the cathode and at colder surfaces in contact with the plasma, which is typically the earth-shield that surrounds the cathode. This process implies that initial quality can also be improved by this method for a-Si based alloys in addition to reduction of SWE in a-Si based solar cells. Combining all these improvements would allow a-Si based solar cells with 4 junctions to reach efficiencies over 20% and make this low EPBT technology viable for widespread adoption.

## Data Availability

The datasets generated and/or analysed during the current study are available in the figshare repository, https://doi.org/10.6084/m9.figshare.23523138.v1.

## References

[1] Fernández, L. Global cumulative installed solar PV capacity 2021. *Statista*. (https://www.statista.com/statistics/280220/global-cumulative-installed-solar-pv-capacity) (2023).

[2] Enkhardt, S. Global solar capacity additions hit 268 GW in 2022. *PV Mag.* December 23. (https://www.pv-magazine.com/2022/12/23/global-solar-capacity-additions-hit-268-gw-in-2022-says-bnef) (2022).

[3] Zieliński, M. *et al*. Ember global electricity review, March 2022. (https://ember-climate.org/insights/research/global-electricity-review-2022) (2022).

[4] Xiong, G., Metzger, W.* Comprehensive Renewable Energy* 2nd Edn Volume 1, p362–387. 10.1016/B978-0-12-819727-1.00137-0 (2022).

[5] Bhandari KP (2015). Energy payback time (EPBT) and return on energy invested (EROI) of solar photovoltaic systems: A systematic review and meta-analysis. Renew. Sustain. Energy Rev..

[6] Virtuani, A. *et al*. Overview of temperature coefficients of different thin film photovoltaic technologies in *5*^*th*^* WCPEC/25*^*th*^* EUPVEC, 4AV.3.83*. 10.4229/25thEUPVSEC2010-4AV.3.83 (2010).

[7] Zammit, M. *et al*. An evaluation of kWh/kWp values as the standard to adequately differentiate between PV technologies, chamber of engineers malta, in *23rd Annual Engineering Conference: Energy and Transportation, Challenges and Opportunities* (2015).

[8] Deng X, Schiff EA, Luque A, Hegedus S (2003). Amorphous silicon based solar cells. Handbook of Photovoltaic Science and Engineering.

[9] Staebler DL, Wronski CR (1977). Reversible conductivity changes in discharge-produced amorphous Si. Appl. Phys. Lett..

[10] Kim S (2013). Remarkable progress in thin-film silicon solar cells using high-efficiency triple-junction technology. Sol. Energy Mater. Sol. Cells.

[11] Yan B (2011). Innovative dual function nc-SiO_x_: H layer leading to a> 16% efficient multi-junction thin-film silicon solar cell. Appl. Phys. Lett..

[12] Isabella O (2014). Thin-film silicon-based quadruple junction solar cells approaching 20% conversion efficiency. Sol. Energy Mater. Sol. Cells.

[13] Guha S (2001). Amorphous semiconductor solar cells. Encyclopedia of Materials Science and Technology.

[14] Ganguly G (1994). Optimization of stabilized performance of amorphous silicon solar cells deposited at high growth rates by de-coupling of gas and substrate temperatures. Appl. Surf. Sci..

[15] Takagi T (1999). Gas-phase diagnostics and high-rate growth of stable a-Si:H. Thin Solid Films.

[16] Becerra R (1995). Prototype Si—H insertion reaction of silylene with silane. Absolute rate constants, temperature dependence, RRKM modelling and the potential-energy surface. J. Chem. Soc. Faraday Trans..

[17] Ganguly, G. *Private Communication* (BP Solar, 3601 LaGrange Pky, 2003) (unpublished).

[18] Van Sark WGJHM (2002). Methods of deposition of hydrogenated amorphous silicon for device applications. Thin Films Nanostruct..

[19] Ganguly G (2003). Effect of Temperature and temperature uniformity on plasma and device stability. Mat. Res. Soc. Symp. Proc..

[20] Matsuda A (1988). Independent control of spin density and hydrogen-bonding configuration in glow discharge-hydrogenated Si-Ge alloys using cathode-heating method. Appl. Phys. Lett..

[21] Banerjee R (1993). Control of powder formation in silane discharge by cathode heating and hydrogen dilution for high-rate deposition of hydrogenated amorphous silicon thin films. J. Appl. Phys..

[22] Ganguly G, Matsuda A (1994). Growth process of a-Si:H. Optoelectron. Devices Technol..

[23] Yue G (2005). Correlation between powder in the plasma and stability of high rate deposited a-Si:H. Mater. Res. Soc. Symp. Proc..

